# Alteration of the colostrum whey proteome in mothers with gestational hypothyroidism

**DOI:** 10.1371/journal.pone.0205987

**Published:** 2018-10-17

**Authors:** Lingli Chen, Jingxuan Wang, Pingping Jiang, Fazheng Ren, Xingen Lei, Huiyuan Guo

**Affiliations:** 1 Beijing Advanced Innovation Center for Food Nutrition and Human Health, College of Food Science and Nutritional Engineering, China Agricultural University, Beijing, China; 2 Key Laboratory of Functional Dairy, co-constructed by Ministry of Education and Beijing Government, China Agricultural University, Beijing, China; 3 Department of Veterinary and Animal Sciences, University of Copenhagen, Frederiksberg, Denmark; 4 Department of Animal Science, Cornell University, Ithaca, NY, United States of America; 5 Hebei Engineering Research Center of Animal Product, Sanhe, China; University of Illinois, UNITED STATES

## Abstract

**Background:**

Gestational hypothyroidism (G-HypoT) is one of the most common thyroid diseases in pregnant women. Human milk, which closely links the mother with infant, is an important factor to the infant health. Here, we analyzed the colostrum whey proteome of women with or without G-HypoT.

**Methods and results:**

Using high–mass accuracy and high-resolution liquid chromatography-tandem mass spectrometry (LC-MS/MS), 1055 proteins were identified. Tandem Mass Tags (TMT) analysis identified differentially expressed proteins between G-HypoT and non-G-HypoT mothers. Of 44 proteins identified, 15 proteins were significantly increased in G-HypoT colostrum whey, while 29 were significantly decreased. Analysis revealed that enzymes involved in carbohydrate metabolism, and that reflect the metabolic activities in breastfeeding women, including fructose-1, 6-bisphosphatase 1, phosphoglycerate mutase 1 were down-regulated. Cell structural proteins, biomarkers of mammary integrity development, including Glyceraldehyde-3-phosphate dehydrogenase (GAPDH) and actin were lower in G-HypoT colostrum whey. However, immune protein fragments like Ig gamma-3 chain C region increased in G-HypoT colostrum whey.

**Conclusion:**

These results implied that G-HypoT may changed human colostrum whey protein in composition level, decreasing levels of metabolic proteins and cell-structure proteins, while increasing levels of immune-related proteins, which may compromise or reflect mothers’ and infants’ health.

## Introduction

Hypothyroidism (HypoT), characterized by an increased level of thyrotropin (TSH) and a decreased level of free thyroxine (T4), affects 3–10% of women; its onset frequently occurs during child-bearing years, triggered by the physiological changes in the thyroid during pregnancy [1–3]. Overt hypothyroidism is defined as TSH concentrations above the reference range and free T4 levels below the reference range, while subclinical hypothyroidism is defined as TSH levels above the reference range when levels of free T4 are within the population reference range [4, 5]. In China, G-HypoT has emerged with an increasing prevalence in pregnant women. Since the clinical symptoms of HypoT, such as fatigue, constipation, cold intolerance, muscle cramps, edema, dry skin, hair loss, and a prolonged relaxation phase of deep tendon reflexes, are easily confused with common signs of pregnancy, therefore they are often ignored by pregnant women [6]. However, the resultant deleterious effects on the mothers themselves and especially their offspring cannot be ignored. Some studies show that gestational HypoT is associated with severe preeclampsia, gestational diabetes, abruptio placentae, a higher incidence of preterm birth, increased fetal mortality, slow weight gain, and impaired cognitive development in offspring [3, 7–9]. Human milk provides an important link between mothers and their infants, and the effects of HypoT on lactation are gaining increasing attention.

Previous studies reported that HypoT directly or indirectly regulates transcription in mammary cells by regulating levels of circulating hormones such as corticosterone, prolactin, and progesterone which can impact the quality and quantity of milk synthesis [10]. This may be attributed to the fact that prolactin promotes mRNA synthesis of milk proteins such as β-casein and α-lactalbumin, and adequate concentrations of thyroid hormones are essential for milk production in response to prolactin [11]. Another report demonstrated that propyl-2-thiouracil-induced HypoT histological changes consistent with early involution of mammary tissue in lactating rats [12]. Motil et al. reported that the plasma thyroxine level of mothers is not only positively correlated with the quantity of milk production, but also affects synthesis of milk protein [13–15]. However, a comprehensive analysis of the breast milk protein profiles of G-HypoT mothers has yet to be reported.

Proteomic technologies could be applied to improving our knowledge of the proteins present in breastmilk. Several studies have characterized the proteome of human breastmilk in different conditions, such as different gestational duration [16], lactation time [17, 18], and health state during lactation [19, 20]. Grapov et al. (2015) leveraged the high mass accuracy, high resolution, and rapid scanning ability of the Q Exactive Orbitrap mass spectrometer to explore the effects of gestational diabetes mellitus (GDM) on lactation and breast milk components. Among the total 601 proteins identified, 260 were quantified using label-free spectral counting. Hettinga et al. (2015) also used a label-free method to identify differences in milk proteins between allergic and non-allergic mothers, and found that 19 of the total 364 proteins identified differed significantly in concentration between the breast milk of allergic and non-allergic mothers [20]. These approaches have greatly advanced our knowledge of milk proteins, but they have not been applied determining the effects of G-HypoT on the proteome of human colostrum. Colostrum is the first best natural food for the newborn, it has more bioactive compounds than mature milk, which lay the foundation of infants immune protection.

In the current study, we isolated whey from colostrum collected from women with or without G-HypoT, and analyzed the proteome using high-resolution, high–mass accuracy liquid chromatography tandem mass spectrometry (LC−MS/MS). To our knowledge, this is the first study to investigate how the proteome of human colostrum is altered by gestational hypothyroidism.

## Materials and methods

### Experimental reagents

Rabbit antibody against complement 4b (C4b), complement factor H (H), human immunoglobulin G (IgG), and mouse antibody against Complement 3 (C3) were obtained from Abcam (Cambridge, UK). Rabbit antibody against α-tubulin, GAPDH, and β-actin were purchased from Cell Signaling Technology (Beverly, MA, USA). β-actin antibodies were obtained from Beyotime (Nantong, China).

### Subject enrollment

Colostrum samples were obtained from women who delivered term infants and were diagnosed with (n = 8) or without G-HypoT (n = 8) at the General Army Hospital of Beijing (Beijing, China). Participants were screened for G-HypoT based on routine obstetrical examination results. Subjects with serum thyrotropin higher than 2.5 mIU/L and serum total thyroxine less than 40 ng/mL in first trimester of pregnancy were diagnosed with G-HypoT, as suggested by the Chinese Society of Endocrinology and the Chinese Society of Perinatal Medicine in 2012. Mothers with G-HypoT received thyroxine replacement therapy during pregnancy. Mothers with maternal illnesses, such as cold, mastitis, or diabetes, were excluded. Written informed consent was obtained from each participant, and this study was approved by the Institutional Review Board (IRB) at the General Army Hospital (No.100). Diet, anthropometric and health history, and status upon collection were obtained from self-reported questionnaires.

### Milk sample collection and processing

Colostrum samples were collected from each breast by hand expression or manual breast pump into 50 mL polypropylene containers within 48 h of lactation initiation [18, 21]. All samples were immediately transported to the lab on ice and stored at −80°C. Samples from each subject were analyzed independently without pooling. As fat and casein disturbs the proteomics analysis, milk samples were thawed and then centrifuged at 10,000 × *g* at 4°C for 10 min to remove the cream layer. Aliquots of skim milk were centrifuged at 100,000 × *g* at 4°C for 60 min to pellet the casein micelles [22]. The transparent milk serum fraction was collected and the final concentration of whey protein was measured by the bicinchoninic acid (BCA) method.

### LC-MS based proteomics with TMT labelling

In-solution digestion was performed according to the procedure described by Chen et al. [23]. Equal amount (100 μg) of whey protein samples was diluted with 8 M urea in PBS and treated with dithiothreitol (DTT, final concentration of 10 mM) for 1 h, and alkylated with iodoacetamide (IAA, 25 mM) for 30 min in the dark. After alkylation, samples were diluted and subjected to in vitro digestion using trypsin (in PBS; 1:100 protease-to-protein ratio)at 37°C overnight. The samples were then desalted using Oasis HLB columns (Waters, MA) and labeled with TMT reagents before LC MS/MS analysis. The S1 File (Supplemental Experimental Procedures) contain details about the MS parameters used for these analyses.

### Data processing strategy and statistical analysis

For each identified protein, the relative abundance in a specifically labelled sample was calculated as the ratio of its abundance in this sample to that in the pooled sample (1055 proteins). Only proteins detected in at least five samples in both groups, G-HypoT and non-G-HypoT, were selected for statistical analysis (472 proteins). Protein relative abundance data and accession numbers were imported into R [24] integrated with R studio [25] for data analysis. Data from three runs were combined following a previously described procedure [26]. Each effect feature was fitted to two linear mixed-effect models with and without the disease condition as the fixed-effect factor, with TMT plex number as a random-effect factor, using the lmer function in the package ‘lme4’ [27]. P-values were generated by comparing these two models with the ANOVA function. GO annotation of these proteins was carried out using the Database for Annotation, Visualization and Integrated Discovery (DAVID) software using the *Homo sapiens* genome as a reference [28, 29]. All differentially expressed proteins between colostral whey from women with versus without G-HypoT were evaluated for GO term enrichment for biological processes, molecular functions, cellular components, and KEGG pathway. The total identified proteins were used as a background set. Significantly overrepresented terms were identified using the hypergeometric test (p-value ≤ 0.05) [30]. Protein interaction networks were analyzed using Search Tool for the Retrieval of Interacting Genes/Proteins (STRING; version 10.0) [31].

### Western blotting

For western blotting, equal amounts of whey protein were separated by SDS-PAGE, followed by transfer of proteins to a polyvinylidene difluoride membrane (Millipore, Temecula, USA). The membrane was incubated with primary antibodies, followed by incubation with horseradish peroxidase-conjugated secondary antibody. The washed blot was detected using chemiluminescence (Amersham ECL™, GE Healthcare, Buenos Aires, Argentina) and quantified by densitometry using QuantityOne (Bio-Rad). Since expression of proteins commonly used for quantitation was affected by HypoT induction, we used a densitometric analysis of the total protein intensity by Ponceau staining as a loading control [32].

## Results

### Number of characterized proteins in human colostrum

Table 1 shows that non-G-HypoT and G-HypoT mothers had very similar demographic characteristics in maternal age, pregnancy weight increase, maternal BMI. All mothers had no mastitis infection. After removal of casein and fat, the aqueous whey fraction of colostrum was obtained. Overall, 1055 proteins were identified in all mothers’ colostrum samples, in triplicate experiments (for details, see S1 Table), and 472 final proteins were used in subsequent statistical and multivariate data analyses.

**Table 1:** Demographics of subjects with and without gestational hypothyroidism.

Characteristics	non-G-HypoT (n = 8)			G-HypoT (n = 8)		
	Mean ± SD	Max.	Min.	Mean ± SD	Max.	Min.
maternal age (y)	31.1 ± 3.9	37.0	26.0	31.5 ± 4.6	37.0	23.0
maternal height (cm)	163.2 ± 5.2	170.0	158.0	165.0 ± 4.9	170.0	160.0
pregnancy weight increase(kg)	17.8 ± 7.3	30.0	5.0	13.3 ± 5.5	22.0	4.5
maternal prepregnancy BMI (kg/m ^2^)	21.5 ± 2.4	24.0	17.3	22.9 ± 3.6	28.2	17.8
maternal pregnancy BMI (kg/m ^2^)	28.1 ± 2.8	30.5	22.3	27.7 ± 4.1	32.0	23.1
gestational age of infant (week)	39.6 ± 1.4	41.1	36.9	38.9 ± 0.7	38.3	40.1
Infant birth length (cm)	51.1±1.1	53.0	50.0	52.40±1.6	55.0	50.0
infant birth weight (kg)	3.4 ± 0.4	4.0	2.9	3.5 ± 0.4	2.9	4.1
Infant jaundice duration (d)	30±17.3 30 7 50±14.1 60 30					
	Frequencies					
primiparous/multiparous		5/3			7/1	
c-section/vaginal		6/2			8/0	
female/male		5/3			3/5	

### Differentially expressed colostral whey proteins between non-G-HypoT and G-HypoT mothers

**Fig 1:**
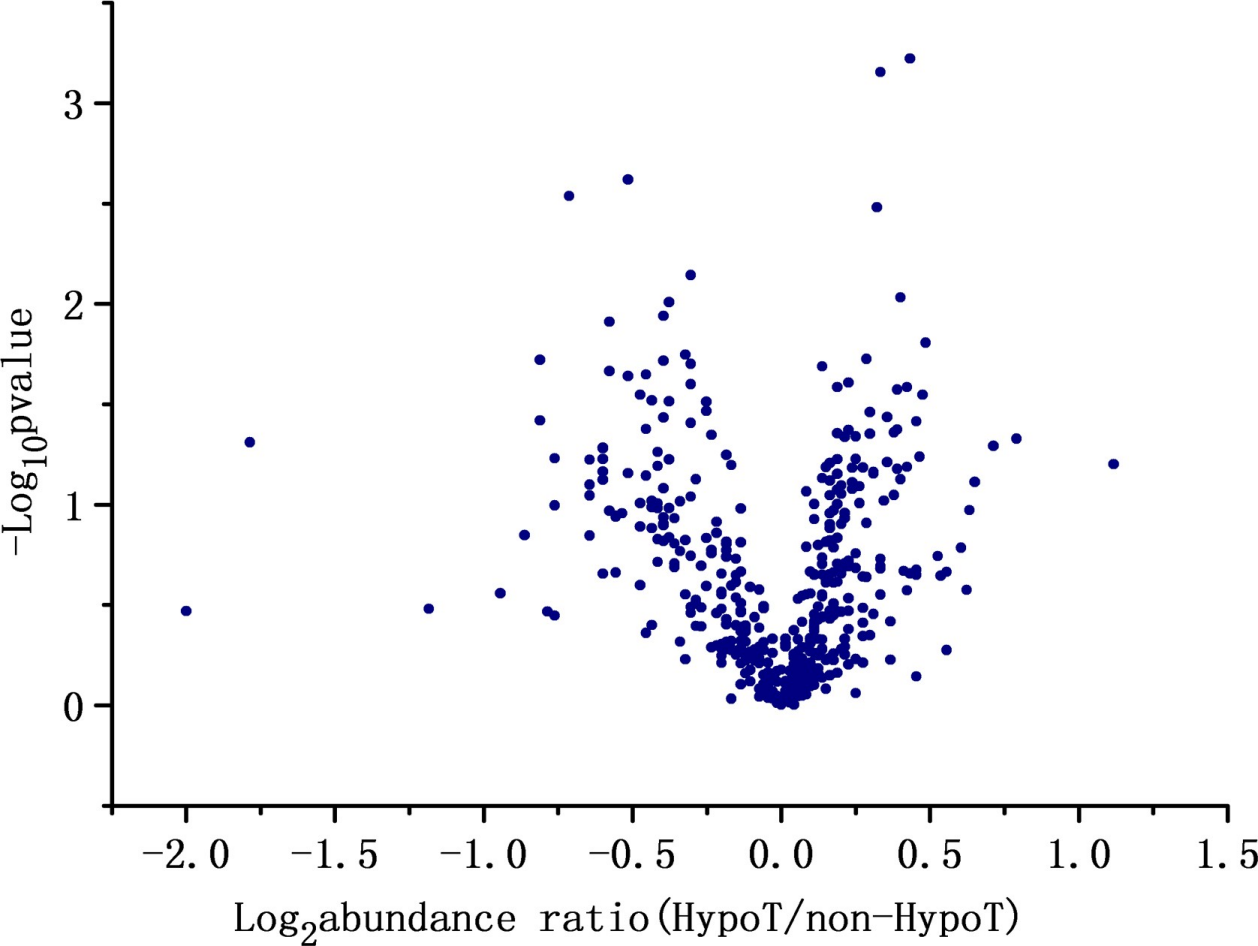
Differences in the proteome of milk from mothers with or without gestational hypothyroidism.

Fig 1 shows the mean log2 ratio of abundance values of the milk from G-HypoT mothers over non-G-HypoT mothers. This volcano figure shows the proteins up-regulated in G-HypoT mothers on the right, and the down-regulated proteins on the left. Statistical analysis indicated that a total of 44 proteins were differentially expressed (for details, see Table 2). Among these, 29 proteins were down-regulated and 15 proteins were up-regulated.

**Table 2:** Differentially expressed proteins in human colostral whey.

Accession	Protein name	Gene name	Biological function	Subcellular function	fold change
	**Down-regulated in G-HypoT**				
P07900	Heat shock protein HSP 90-alpha	HSP90AA	Signaling transduction	Cytoplasm	-1.31
Q99832-3	Isoform 3 of T-complex protein 1 subunit eta	CCT7	Protein metabolism	Cytoplasm	-1.4
P60709	Actin, cytoplasmic 1	ACTB	Cell motility	Cytoplasm	-1.33
P63267	Actin, gamma-enteric smooth muscle	ACTG2	Cell motility	Cytoplasm	-1.44
Q15293-2	Isoform 2 of Reticulocalbin-1	RCN1	Lipid metabolism	ER	-1.24
P13639	Elongation factor 2	EEF2	Protein metabolism	Cytoplasm	-1.3
P32119	Peroxiredoxin-2	PRDX2	Redox response	Cytoplasm	-1.25
P60953	Cell division control protein 42 homolog	CDC42	Cell motility	Cytoplasm	-1.24
P06733	Alpha-enolase	ENO1	Carbohydrate Metabolism	Cytoplasm	-1.33
P18669	Phosphoglycerate mutase 1	PGAM1	Carbohydrate Metabolism	Cytoplasm	-1.28
P07737	Profilin-1 OS = Homo sapiens	PFN1	Cell motility	Cytoplasm	-1.44
P60174-1	Isoform 2 of Triosephosphate isomerase	TPI1	Carbohydrate Metabolism	Cytoplasm	-1.25
Q8NC51-4	Isoform 4 of Plasminogen activator inhibitor 1 RNA-binding protein	SERBP1	Nucleic acid metabolism	Cytoplasm	-1.23
H3BRU6	Poly(rC)-binding protein 2 (Fragment)	PCBP2	Nucleic acid metabolism	Unclear	-1.29
P09467	Fructose-1,6-bisphosphatase 1	FBP1	Carbohydrate metabolism	Cytoplasm	-1.31
P68032	Actin, alpha cardiac muscle 1	ACTC1	Cell motility	Cytoplasm	-1.35
Q16851	UTP—glucose-1-phosphate uridylyltransferase	UGP2	Nucleic acid metabolism	Cytoplasm	-1.36
P23528	Cofilin-1	CFL1	Cell motility	Cytoplasm	-1.34
Q96KP4	Cytosolic non-specific dipeptidase	CNDP2	Protein metabolism	Cytoplasm	-1.23
A0A075B6I9	Protein IGLV7-46 (Fragment)	IGLV7-46	Unclear	Unclear	-1.26
E7EQG2	Eukaryotic initiation factor 4A-II	EIF4A2	Nucleic acid metabolism	Unclear	-1.27
J3KPD9	Nucleoside diphosphate kinase B	NME2	Signaling transduction	Unclear	-1.25
P04406	Glyceraldehyde-3-phosphate dehydrogenase	GAPDH	Carbohydrate Metabolism	Cytoplasm	-1.34
P62937	Peptidyl-prolyl cis-trans isomerase A	PPIA	Protein metabolism	Cytoplasm	-1.26
J3KNQ2	Fibronectin type III domain-containing protein 1 (Fragment)	FNDC1	Unclear	Unclear	-1.72
P07195	L-lactate dehydrogenase B chain	LDHB	Carbohydrate Metabolism	Cytoplasm	-1.35
P30086	Phosphatidylethanolamine-binding protein 1	PEBP1	Signaling transduction	Cytoplasm	-1.28
G3V1A4	Cofilin 1 (Non-muscle), isoform CRA_a	CFL1	Cell motility	Cytoplasm	-1.31
P29401	Transketolase	TKT	Carbohydrate metabolism	Nucleus	-1.37
	**Up-regulated in G-HypoT**				
B1AMI1	ATP-binding cassette sub-family A member 1	ABCA1	Unclear	Unclear	1.35
Q16651	Prostasin	PRSS8	Unclear	Secreted	1.27
A0A087WVJ0	Mucin-1 (Fragment)	MUC1	Protein metabolism	Membrane	1.32
E9PNW4	CD59 glycoprotein	CD59	Immunity	Unclear	1.25
H3BTQ8	Kunitz-type protease inhibitor 1 (Fragment)	SPINT1	Unclear	Unclear	1.22
P07996	Thrombospondin-1	THBS1	Immunity	ER	1.35
K7EK07	Histone H3 (Fragment)	H3F3B	Unclear	Nucleus	1.41
P04003	C4b-binding protein alpha chain	C4BPA	Immunity	Secreted	1.35
A0A075B6K5	HCG2043239 (Fragment)	IGLV3-9	Unclear	Unclear	1.74
Q5VZR0	Golgi-associated plant pathogenesis-related protein 1	GLIPR2	Unclear	Secreted	1.4
F5H2L1	G-protein-coupled receptor 126 (Fragment)	GPR126	Unclear	Unclear	1.23
P01608	Ig kappa chain V-I region Roy		Immunity	Secreted	1.64
P25311	Zinc-alpha-2-glycoprotein	AZGP1	Immunity	Secreted	1.31
A0A087WXL8	Ig gamma-3 chain C region	IGHG3	Immunity	Unclear	1.29
Q13410	Butyrophilin subfamily 1 member A1	BTN1A1	Lipid metabolism	Secreted	1.24

### Biological functions and subcellular localization of identified proteins

**Fig 2:**
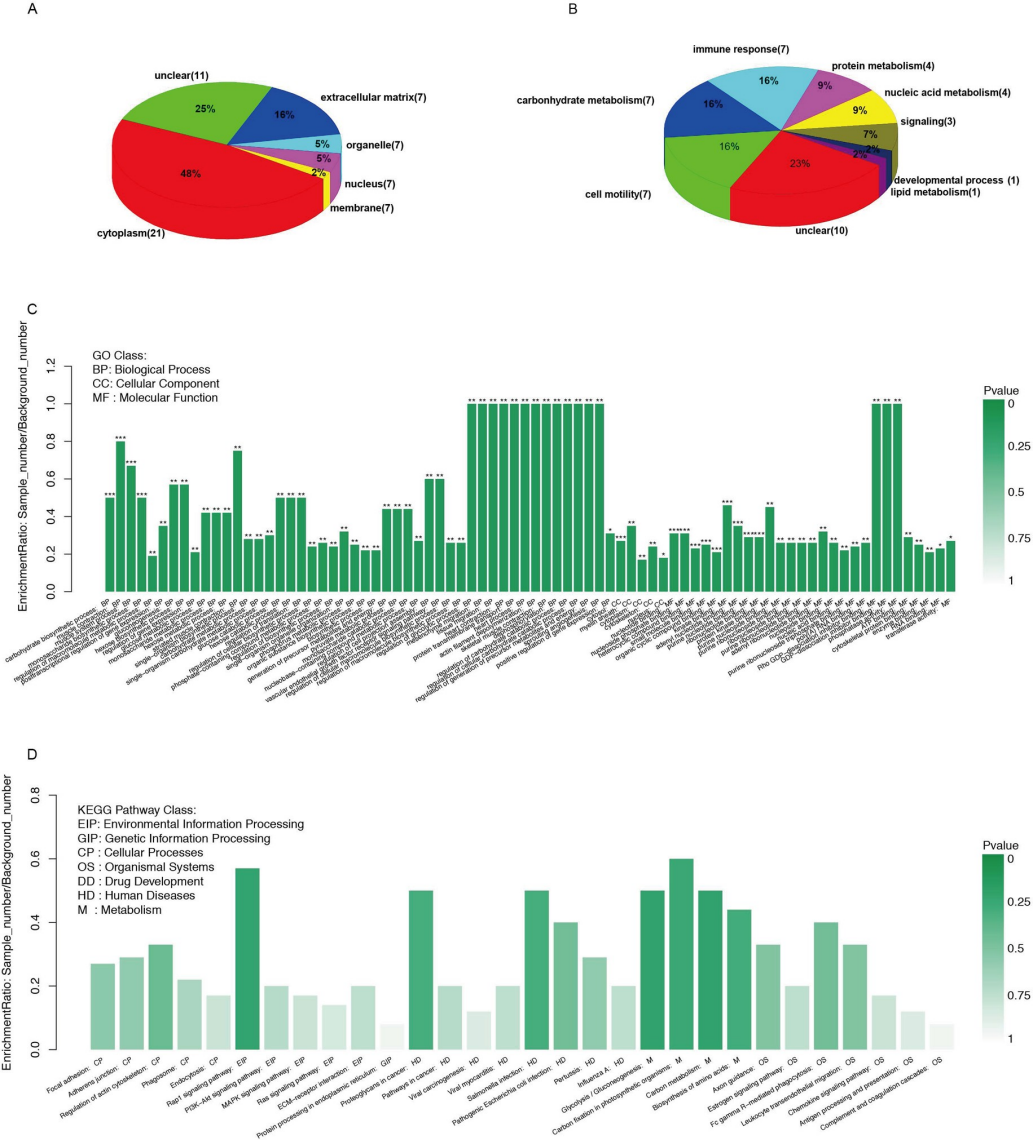
Different protein classifications and western blot validation. (A) Functional categorization of 44 proteins identified in the significance analyses. (B) Subcellular location of all 44 proteins identified in the significance analyses. The number of proteins in each category is indicated in parentheses. Enrichment analysis of differentially expressed proteins (C) Gene ontology (D) KEGG pathway.

The 44 proteins were categorized by both their subcellular location and their function based on annotations in the UniProt Database. Fig 2A shows that the majority of the differentially expressed proteins were cytoplasmic (48%), and only 16% were secreted proteins that function in the extracellular space. Proteins originating from organelles, the nucleus, and the membrane accounted for approximately 12% of the total; the subcellular location of approximately 25% of proteins was unclear.

As shown in Fig 2B, metabolism-related proteins appeared to be the main biological function group (36%), especially carbohydrate metabolism. Cytoskeletal proteins accounted for 16%, and 7 of the 44 differentially expressed proteins were involved in immune response. No clear GO annotation was available for 23% of proteins, which makes it difficult to clarify their relationship with G-HypoT mothers and/or infants.

### Gene ontology and KEGG pathway enrichment analysis

Fig 2C shows that 44 altered proteins in G-HypoT were enriched (p < 0.05) in gene ontology (GO) for many terms for biological processes, molecular functions, and cellular components. Among biological processes, the most significantly (p < 0.001) enriched terms were GO: 0016051 (carbohydrate biosynthetic process), GO: 0006936 (muscle contraction), GO: 0003012 (muscle system process), and GO: 0046364 (monosaccharide biosynthetic process). These can be summarized as muscle systems and carbohydrate metabolism. Among cellular components, the most significantly (p < 0.001) enriched term was GO: 0005829 (cytosol). Among molecular functions, nine terms were significantly (p < 0.001) enriched, all associated with compound binding. Fig 2D shows the results of KEGG pathway analysis, with darker colors indicating deeper KEGG enrichment; however, no KEGG pathways were significantly enriched.

### Hierarchical cluster analysis

**Fig 3:**
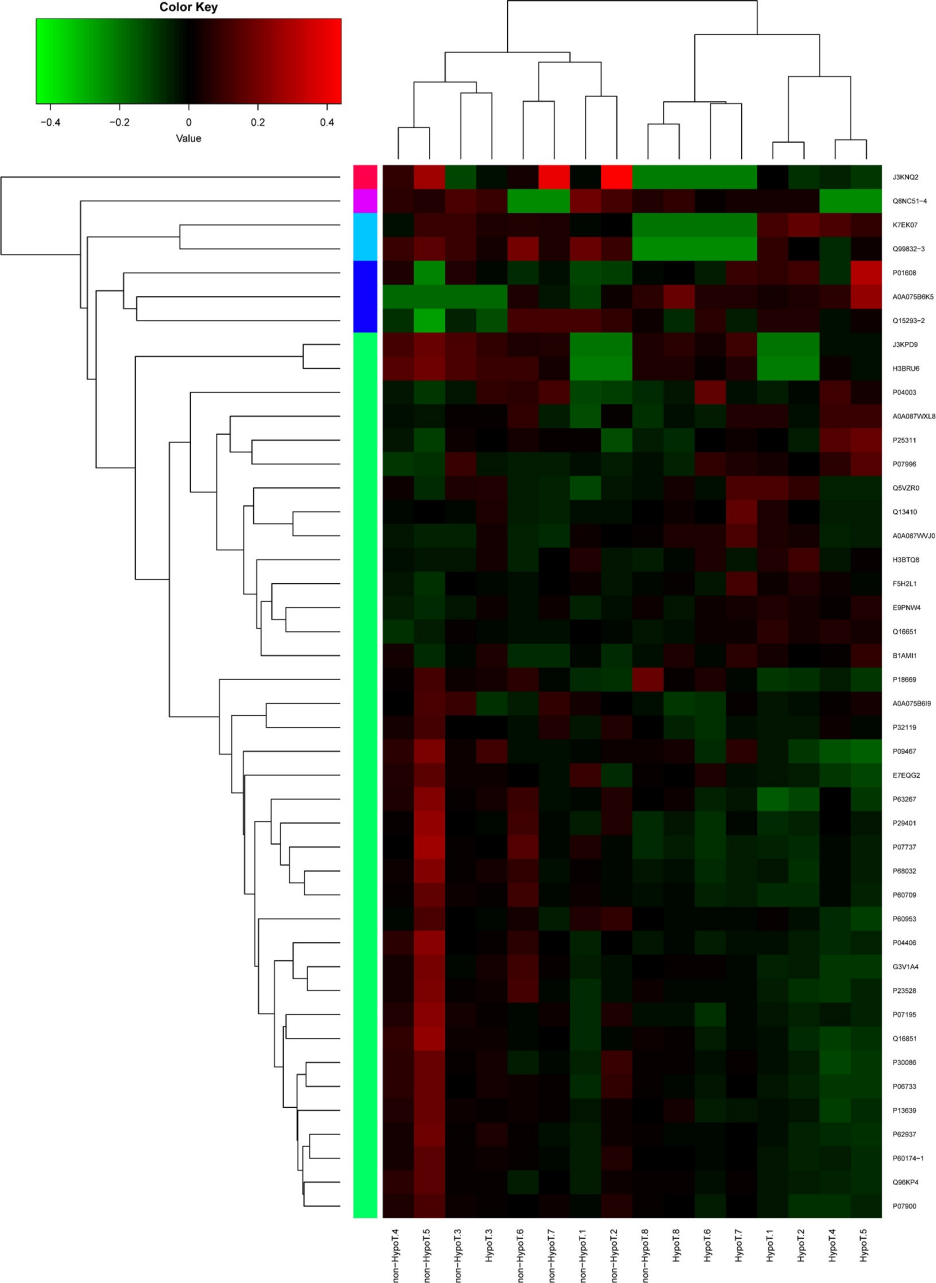
Hierarchical clustering of differentially expressed colostral whey proteins between mothers with or without gestational hypothyroidism. Bar color represents a logarithmic scale from −0.4 to 0.4. Red indicates higher expression and green indicates lower expression.

**Fig 4:**
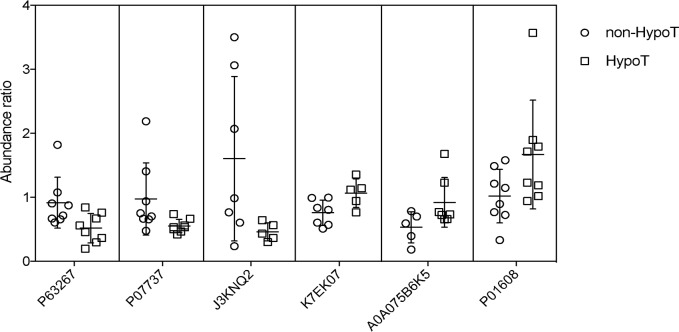
Abundance for six proteins in individual colostrum samples from mothers with or without gestational hypothyroidism.

In Fig 3, each column contains differentially expressed proteins clustered by individual mother and each row contains individual differentially expressed proteins clustered between the two groups. Red text indicates higher expression and green text indicates lower expression. Except in two mothers, which may be attributed to individual differences, milk from G-HypoT and non-G-HypoT mothers showed two distinct cluster patterns. As shown in Fig 3, non-G-HypoT mothers clustered mainly to the left, whereas G-HypoT mothers clustered to the right. Most of the up-regulated proteins were higher in G-HypoT colostrum (red), such as immune-related protein P01608 (Ig kappa chain V-I region Roy) and A0A087WXL8 (Ig gamma-3 chain C region) (Table 2). Some proteins were also down-regulated in G-HypoT mothers, shown in green, such as the cytoskeletal proteins actin, tubulin, and GAPDH. Thus, the proteomic differences between the two groups were mainly due to their concentration instead of the presence or absence of specific proteins. To visualize whether these differences in protein intensity were due to a few outliers or consistent differences between the groups, the data for individual mothers for the six proteins that showed the largest differences, P63267 (actin, gamma-enteric smooth muscle), P07737 (profilin-1 OS = Homo sapiens), J3KNQ2 (fibronectin type III domain-containing protein 1 (Fragment)), K7EK07 (histone H3 (Fragment)), A0A075B6K5 (HCG2043239 (Fragment)), and P01608 (Ig kappa chain V-I region Roy), were plotted. Fig 4 shows that for these six proteins, the significant differences were due to systematic differences between groups, not outliers.

### Western blot validation

**Fig 5:**
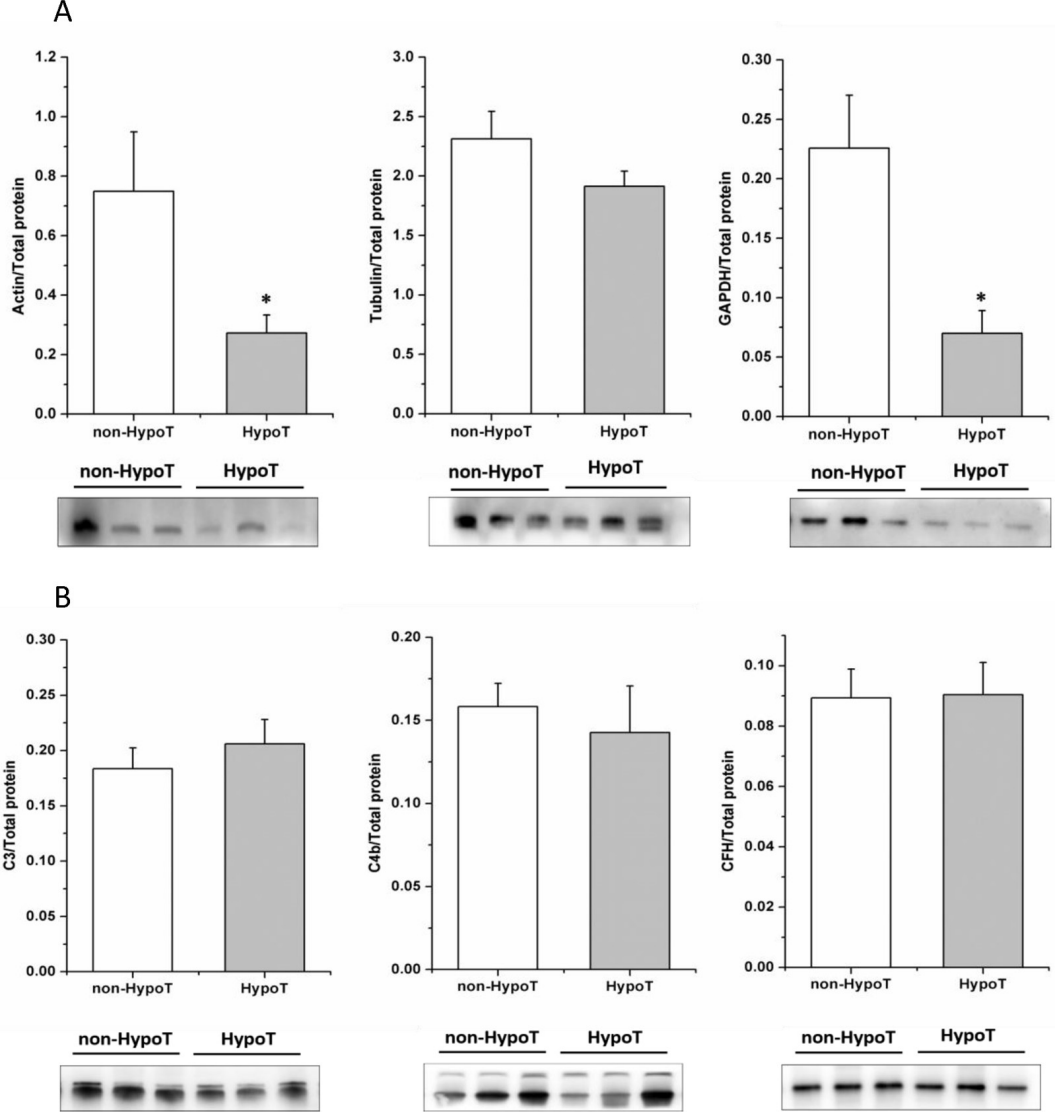
Western blot validation. (A) cell structural proteins between two groups from three individual donors. (B) complement proteins between two groups from three individual donors. C3, complement C3; C4b, complement C4b; CFH, complement factor H.

Some cell structural proteins, such as actin and GAPDH, showed lower expression in G-HypoT milk. These proteins are known to be housekeeping proteins, and their expression is typically stable. We validated this result using western blotting. Fig 5A shows that actin, tubulin, and GAPDH were indeed down-regulated in G-HypoT mothers’ milk.

Immune proteins in human breast milk are known to play a critical role in protecting infants from disease [22]. Our results showed that some immune globulins were highly expressed in colostral whey from G-HypoT women compared to non-G-HypoT women, such as P01608 (Ig kappa chain V-I region Roy) and P25311 (Ig gamma-3 chain C region), which are domains of immunoglobulins. Another important immune system is the complement system, which is activated by two major pathways, the classical pathway and the alternative pathway. We wondered whether complement proteins were also affected by G-HypoT. We measured levels of the classical pathway and the alternative pathway characteristic components C3 and CFH (complement factor H), as well as C4b, a key protein involved in the first step of the complement cascade, by western blotting. However, they showed no significant differences (Fig 5B).

### Protein-protein interaction network

**Fig 6:**
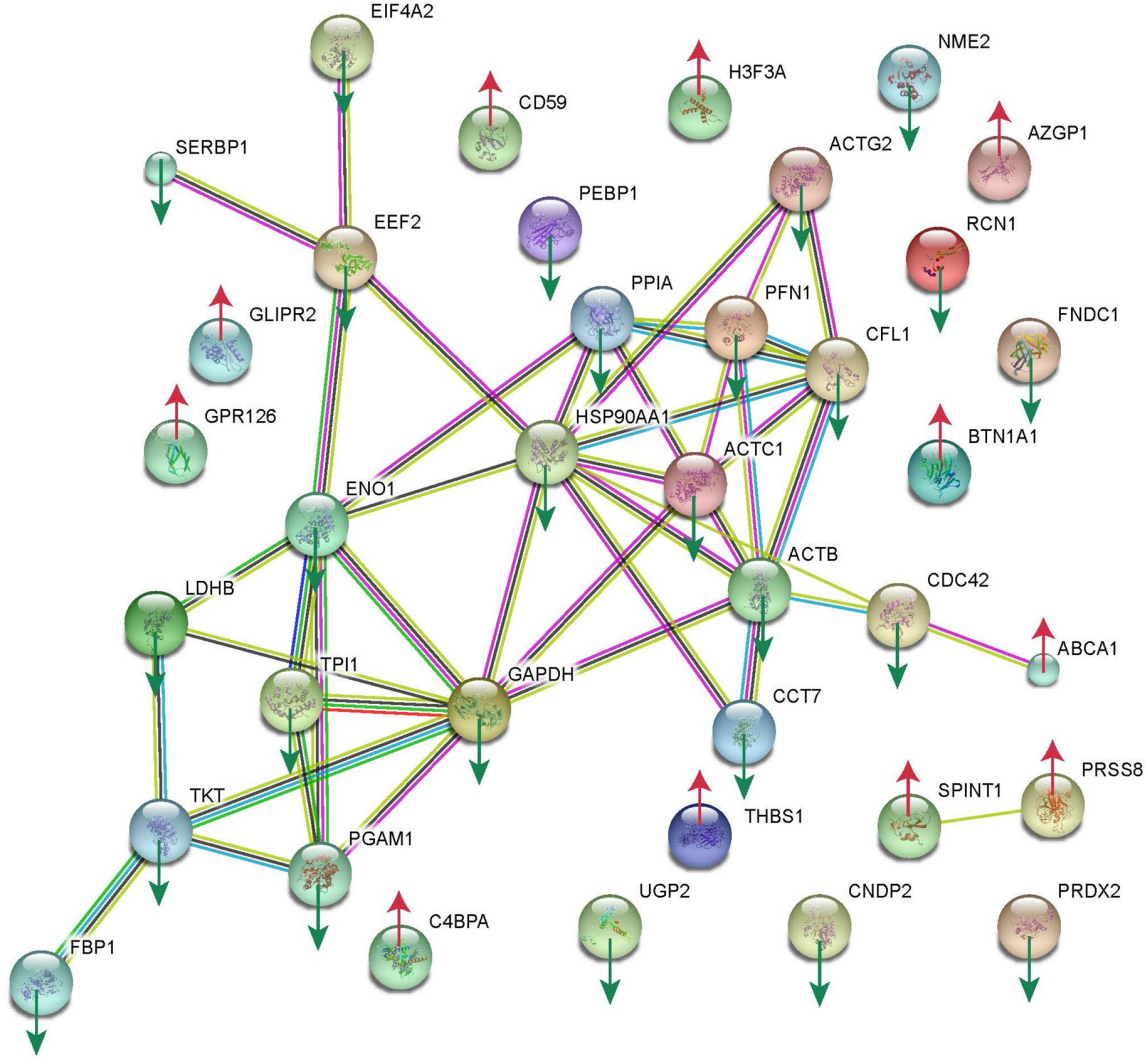
Protein−protein interaction network for differentially colostral whey proteins between mothers with or without gestational hypothyroidism. Proteins up-regulated in the G-HypoT group were labeled with up red arrows, and down-regulated proteins were labeled with down blue arrows.

Analysis of the protein-protein interaction network was conducted using STRING (Fig 6). Thirty-seven of the 44 differentially expressed proteins were directly correlated, 11 of which were up-regulated and 26 of which were down-regulated. Down-regulated proteins GAPDH, Actin, alpha cardiac muscle 1 (ACTC1), Actin, cytoplasmic 1 (ACTB)(cytoskeletal-related) and Alpha-enolase (ENO1), Isoform 2 of Triosephosphate isomerase (TPI1), Phosphoglycerate mutase 1 (PGAM1), L-lactate dehydrogenase B chain (LDHB), Transketolase (TKT) (carbohydrate metabolism related) were closely associated with each other. They may have interactions in expression and play a critical role in the network.

## Discussion

### Overview of the colostrum whey proteome

We annotated a total of 1055 proteins from the whey fraction of human breastmilk using a TMT-labeling proteomic approach. We identified a total of 44 differentially expressed proteins (for details, see Table 2); of these 29 were down-regulated and 15 were up-regulated in G-HypoT. These proteins were associated with three major functional classes: carbohydrate metabolism (down-regulated), cytoskeleton (down-regulated), and immunity (up-regulated) (Fig 6).

### Energy metabolism is modulated in the milk of G-HypoT mothers

Differentially expressed proteins involved in carbohydrate metabolism, protein metabolism, and lipid metabolism were uniformly lower in G-HypoT mothers. The proteins alpha-enolase (P06733), phosphoglycerate mutase 1 (P18669), isoform 2 of triosephosphate isomerase (P60174-1), fructose-1, 6-bisphosphatase 1 (P09467), glyceraldehyde-3-phosphate dehydrogenase (P04406), L-lactate dehydrogenase B chain (P07195), and transketolase (P29401) are all enzymes that participate in carbohydrate metabolism, performing functions such as gluconeogenesis and glycolysis. It is known that the processes and pathways mediating the metabolism of carbohydrates, lipids, and proteins are all affected by thyroid hormones (THs) in almost all tissues [33]. Thus, changes in thyrotropin and/or thyroxine (T4) levels will affect the metabolic processes of G-HypoT mothers. As thyroid hormones are galactopoietic and help to establish the mammary gland metabolic priority during lactation, pregnancy needs mobilize this hormone to make preparation for laction. So it may need more thyroid hormones during pregnancy, this may aggravates a state of clinical or subclinical hypothyroidism, and adequate function of the mammary glands may be compromised [11]. One of the most marked consequences of maternal hypothyroidism on the offspring is stunted growth and delayed maturation of the newborn that leads to mental retardation and subnormal height [10]. Newborn infants rely on their mothers’ milk to obtain nutrition and energy to meet the needs of fast growth.

Fig 6 shows that several enzymes related to carbohydrate metabolism were down-regulated, including GAPDH, ENO1, TPI1, PGAM1, LDHB, and TKT. Glyceraldehyde-3-phosphate dehydrogenase (GAPDH) is a key enzyme in glycolysis that catalyzes the first step of the pathway by converting D-glyceraldehyde 3-phosphate (G3P) into 3-phospho-D-glyceroyl phosphate. ENO1 (alpha-enolase) is involved in the subpathway that synthesizes pyruvate from D-glyceraldehyde 3-phosphate. TPI1 (triosephosphate isomerase) is involved in the gluconeogenesis pathway, catalyzing conversion of D-glyceraldehyde 3-phosphate to glycerone phosphate. PGAM1 (phosphoglycerate mutase 1) is the primer in the interconversion of 3- and 2-phosphoglycerate with 2, 3-bisphosphoglycerate. LDHB (L-lactate dehydrogenase B chain) is involved in the subpathway that synthesizes (S)-lactate from pyruvate. TKT (transketolase) catalyzes the transfer of a two-carbon ketol group from a ketose donor to an aldose acceptor, via a covalent intermediate, with the cofactor thiamine pyrophosphate. Carbohydrates are a vital source of energy, and a lower level of carbohydrate metabolism proteins in the colostrum reflects a weakened metabolic status in mothers. This may aggravate G-HypoT mothers’ discomfort, increasing anemia, fatigue, anorexia, or a combination of these conditions. While we had no date to show the hormonal condition influence on the laction time and milk volume, further study need take this into account. ENO1, TPI1, PGAM1, LDHB, and TKT are carbohydrate metabolism enzymes, most of which are involved in glycolysis of the energy production pathway. The physiological function of TPI1 is to maintain the homeostasis between dihydroxyacetone phosphate and glyceraldehyde-3-phos-phate produced by aldolase in glycolysis, which is interconnected to the pentose phosphate pathway and to lipid metabolism via triosephosphates [34]. Infant dietary fat intake is very high due to their high energy requirement and beneficial effects of fat on growth and development of the brain and vision. Approximately 50% of the total energy intake is acquired from milk lipids during the first month after birth, and body fat accounts for 35% of an infant’s weight gain during the first 6 months [35, 36, 37]. Milk contains enzymes that help to balance three major nutrients transformation, especially lipid synthesis and breakdown [37]. So down-regulated energy metabolism enzymes may influence infants’ energy requirement. Another critical aspect, infants’ gastro-intestinal tracts are immature, changes in carbohydrate metabolism enzymes, which are supposed to be relatively stable during gastric digestion, may affect the gastroenteric function of baby [38]. So a reduction in enzymes may cause unbalanced digestion and absorption, which would place a burden on the infant and influence their growth. We found infant duration of jaundice was longer in G-HypoT (50±14.1) than no-G-HypoT(30±17.3). As G-HypoT mothers underwent thyroxine therapy, it would improve the G-HypoT thyroxine levels. We thought G-HypoT would result in a comprehensive disorder of the whole physical state. The medication therapy improves the thyroxine levels in hypothyroidism state, but may not be able to let patients recovery completely from the disorder state. So whether the therapy controlled levels of thyroid hormones optimally or not, the G-HypoT mother whole physiological state may not healthy as the non-G-HypoT to some degree. But therapy may decrease the differences in colostrum proteome between G-HypoT and no-G-HypoT, thus decrease the influence on the baby and mother, as Table 1 showed no difference in the infant birth weight (3.4 ± 0.4 kg in non-G-HypoT VS 3.5 ± 0.4 kg in G-HypoT) and length (51.1±1.1 cm in non-G-HypoT VS 52.40±1.6 cm in G-HypoT). A large and long-period prospective cohort studies may show obvious phenotypes in infants and/or mothers. Of course, we need further deliberate study to clarify if and/or how therapy affect the proteome changes.

We also detected lower levels of proteins involved in protein metabolism in G-HypoT mothers’ milk, including some enzymes related to protein synthesis. In accordance with thyroid hormones can increase protein synthesis in organ culture [39], hypothyroidism may reduce protein production in mammary glands, as shown in our results.

### Cytoskeletal proteins were down regulated in colostrum of mothers with G-HypoT

Cytoskeletal proteins are often used as loading controls for western blot analysis, due to their usual, stable expression Nevertheless in this study, cell structural proteins were all down-regulated in G-HypoT mothers’ milk (Fig 6). Western blot analysis of actin, tubulin, and GAPDH verified the proteomic results (Fig 5A). β-Actin, α-tubulin, and lamin B expression, indicative of the activation of apoptotic pathways and tissue remodeling, are strongly increased in G-HypoT lactating rats mammary glands[12]. Sokolowski et al. proposed that caspase activation leads to the breakdown of cytoskeletal actin and tubulin dimers [40]. This may brought about an increase in the dynamics of these proteins in mammary glands, along with the initiation of tissue remodeling, ultimately leading to pre-lactational mammary tissue architecture involution. Cell self-repair regulation may locked these proteins in mammary gland for rebuilding cell cytoskeleton and premature involution of mammary glands impairs milk protein production including cytoskeletal proteins. This induces a decrease in the relative abundance of milk cytoskeletal proteins together, as shown in our results.

### G-HypoT changed immune protein levels in colostrum

Human milk is a primary source of immune protection for newborns. Our results showed that expression of six out of seven immune-related proteins was higher in colostral whey from G-HypoT women compared to non-G-HypoT women. P01608 (Ig kappa chain V-I region Roy) and P25311 (Ig gamma-3 chain C region) are domains of immunoglobulins. While the increasing Ig gamma is the light chain of IgG, G-HypoT, as a pathological state, may stimulated the mother IgG production to compensate weaker immunity. In production of colostrum, immunoglobulins pass through mammary epithelial cells from the interstitial spaces between them. The tight junctions of mammary epithelial cells develop leaks during colostrum production. But during mature lactation, immunoglobulins can only enter the milk via transcytosis across mammary epithelial cells. This means that the degree of differentiation and integrity of mammary epithelial cells play a key role in entry of immunoglobulins into milk. Campo Verde Arbocco et al. reported that lactating G-HypoT rats have premature mammary involution, with more debris in the lumen of the alveoli and significantly decreased percentages of total and active alveoli in mammary gland morphology [12]. In Fig 5A, cytoskeletal proteins were decreased in G-HypoT colostrum, reflecting defects in the integrity of mammary glands, which may have permitted increased leakage of immunoglobulins into milk. As discussed above, some immune-related proteins were significantly higher in the milk of G-HypoT mothers (Table 2).

The complement system, another important immune component in human milk, was also different between G-HypoT and non-G-HypoT. As the humoral backbone of the innate immune defense system, the complement system has three converging enzymatic cascades: the classical pathway, the alternative pathway and the lectin pathway [41]. The complement system pathway is a complex cascade reaction involving different components. The final goal is to construct molecular tags (C4b and C3b) that interact with complement receptors, facilitating engulfment of target cells. The complement system is important to control of bacterial and viral infection. Newborns do not possess mature immune capability, and they rely on human milk, especially colostrum, to obtain protection. CD59 was higher in colostrum of G-HypoT mothers. CD59 is a regulator, which acts as an inhibitor of complement-mediated cytolysis, and increased levels of CD59 may reduce infants’ ability to resist pathogens. The main components C3, C4b, and the regulator CFH were detected in both groups, with no significant difference between the two groups (Fig 5B).

### Other altered proteins

Peroxiredoxin-2, which is involved in oxidative stress regulation, protecting cells, enzymes, and other proteins from oxidative damage, was also down-regulated in G-HypoT mothers [42, 43]. Isoform 2 of reticulocalbin-1 (Rcn1), a calcium-binding protein that is involved in the biological process of camera-type eye development and in utero embryonic development, was also down-regulated. It belongs to the family of GREC proteins that are characterized as calcium-binding protein in the endoplasmic reticulum (ER) with EF hands (multiple helix-loop-helix motifs with high affinity for Ca^2+^ binding) [44]. Rcn1 is widely expressed in various fetal and adult organs, and is present in ependymal cells, neuroblasts, and a minority of glial cells in the fetal brain [45]. Although the functional properties of the members of the CREC family are largely unknown, the members of this family are highly conserved may also in the milk, implying important roles in the maintenance of normal cell behavior [44]. Decreased expression may have a negative effect on infants and/or mothers. These proteins are meaningful targets for further investigation, although it is unclear whether they are functionally active in the infant gut. A major milk mucin, MUC1, which is a signaling molecule, was up-regulated in milk of mothers with G-HypoT. As MUC1 is a tumor antigen and oncoprotein that is overexpressed in most tumors, including breast, pancreatic, ovarian, and colon cancers, our result suggests that hypothyroidism may induce abnormal cell metabolism and proliferation of mammary gland cells [46]. This protein should also be considered for its effects on the health of pregnant women. The MUC1 inhibits binding of S-fimbriated Escherichia coli to buccal epithelial cells, it provides protection for infant [47]. The up-regulated MUC1 may be a negative-feedback of weak immunity in both mothers and infants.

## Conclusions

In this observational study, we identified a total of 1055 proteins in human colostrum and found that 44 proteins were differentially expressed in the colostral whey proteome between mothers with and without G-HypoT. G-HypoT possible influenced the human whey proteome of colostrum, decreasing metabolic and cell-structure proteins and increasing immunity-related proteins, which may affect the transportation of nutrition and bioactive components for infants. We found that theduration of jaundice was longer in infants of G-HypoT (50±14.1) mothers than no-G-HypoT(30±17.3) mothers. But there was no difference in infant birth length and weight between the two groups. May be a more large sample will show difference, or therapy relief hypothroidism influence on mothers and thus the milk. So influence of alteration caused by G-HypoT may need a period to make difference on clinical characteristics of infant health. Since our sample size was small, large prospective cohort studies should be performed to verify our findings. The impact of maternal gestational hypothyroidism on infant growth index and nutrient status should also be taken into account, along with changes in milk components during the course of lactation. The present study provides insight into proteomic differences in the colostral whey of G-HypoT women in comparison to non-G-HypoT women.

## Supporting information

S1 File Supplemental experimental procedures. (DOCX)

S1 Table Overview of all identified protein. (XLSX)
