# Supplementary material for: Alteration of the colostrum whey proteome in mothers with gestational hypothyroidism
Source: PLoS One. 2018 Oct 17;13(10):e0205987. doi: 10.1371/journal.pone.0205987 (PMC6192644; doi:10.1371/journal.pone.0205987)
Supplement: S1 File — (DOCX) [file pone.0205987.s001.docx]

**Supplemental Experimental Procedures**

**LC-MS based proteomics with TMT labelling**

Peptides from different samples were labeled with tandem mass tag (TMT) reagents (Thermo, Pierce Biotechnology) according to the manufacturer’s instructions. Briefly, the TMT reagents were dissolved in acetonitrile and added to the peptide solution. The reaction was kept at 25 °C for 1 h, then quenched by 5% hydroxylamine for 15 min. The TMT-labeled peptides were mixed and desalted by HLB column. The peptides were fractionated by a UPLC3000 system (Dionex, CA) with an XBridge BEH300 C18 column (Waters, MA). Mobile phase A was H_2_O with ammonium hydroxide (pH 10), and mobile phase B was acetonitrile in ammonium hydroxide (pH 10). Peptides were separated with the following gradients: 8% to 18% phase B, 30 min; 18% to 32% phase B, 22 min. A total of 48 fractions were collected, dried in a speedvac, combined into 12 fractions, and redissolved in 0.1% formic acid (Chen et al., 2015).

For quantitative proteomic analysis, the TMT-labeled peptides were separated using a120-min gradient elution at a flow rate of 0.30 μL/min with an EASY-nLCII integrated nano-HPLC system (Proxeon, Denmark), which was directly interfaced with a Q Exactive mass spectrometer (Thermo). The analytical column was a fused silica capillary column (75 μm ID, 150 mm length; packed with C-18 resin, Lexington, MA). Mobile phase A consisted of 0.05% formic acid and 2% acetonitrile, and mobile phase B consisted of 98% acetonitrile. The Q Exactive mass spectrometer was operated in the data-dependent acquisition mode using the Xcalibur 2.1.3 software and there was a single full-scan mass spectrum in the Orbi-trap (300–1800 m/z, 70 000 resolution) with automatic gain control (AGC) target value of 3e6. A data-dependent acquisition method was performed to collect generated MS/MS spectra at a resolution of 17500 with an AGC target of 1e5 and a maximum injection time (IT) of 60 ms for the top 10 ions observed in each mass spectrum. The isolation window was set at 2 Da width, the dynamic exclusion time was 60 s and the normalized collisional energy (NCE) was set at 30.

The generated MS/MS spectra were searched against the Human Uniprot database (http://www.uniprot.org/uniprot/?query = taxonomy: 9606) using the SEQUEST searching engine of the Proteome Discoverer (PD) software (version 1.4). The search criteria were as follows: full tryptic specificity was required; one missed cleavage was allowed; carbamidomethylation (C) and TMT sixplex (K and N-terminal) were set as the fixed modifications; the oxidation (M) was set as the variable modification; precursor ion mass tolerances were set at 10 ppm for all MS acquired in an orbitrap mass analyzer; and the fragmentation mass tolerance was set at 20 mmu for all MS2 spectra acquired. The peptide false discovery rate was calculated using Percolator provided by PD. When the q value was smaller than 1%, the peptide spectrum match was considered to be correct. False discovery was determined based on peptide spectrum match when searched against the reverse, decoy database. Peptides only assigned to a given protein group were considered as unique. The false discovery rate was also set to 0.01 for protein identification. Relative protein quantification was performed using Proteome Discoverer software (Version 1.4) according to the manufacturer’s instructions on the six reporter ion intensities per peptide. Quantitation was carried out only for proteins with one or more unique peptide matches. Protein ratios were calculated as the median of all peptide hits belonging to a protein. All known contaminants (i.e. keratins, trypsin), and proteins detected in less than 60% of the samples, were removed from the set of proteins identified. Quantitative precision was expressed as protein ratio variability (Chen et al., 2015).

**SUPPLEMENTAL REFERENCES**

[1] Chen, Y. L., Yang, F., Sun, Z. Y., Wang, Q. T., *et al.,* Proteomic Analysis of drug-resistant Mycobacteria: Co-evolution of copper and INH Resistance. *Plos One* 2015, *10(6)*, e0127788.
